# Toughening Mechanisms in Nanolayered MAX Phase Ceramics—A Review

**DOI:** 10.3390/ma10040366

**Published:** 2017-03-30

**Authors:** Xinhua Chen, Guoping Bei

**Affiliations:** 1 School of Mechanical Electronic and Automobile Engineering, Beijing University of Civil Engineering and Architecture, Beijing 100044, China; chenxinhua@bucea.edu.cn; 2 Department of Materials Science and Engineering, 3ME, Delft University of Technology, Mekelweg 2, 2628CD Delft, The Netherlands

**Keywords:** cracks, toughening mechanism, MAX phase, finite element model

## Abstract

Advanced engineering and functional ceramics are sensitive to damage cracks, which delay the wide applications of these materials in various fields. Ceramic composites with enhanced fracture toughness may trigger a paradigm for design and application of the brittle components. This paper reviews the toughening mechanisms for the nanolayered MAX phase ceramics. The main toughening mechanisms for these ternary compounds were controlled by particle toughening, phase-transformation toughening and fiber-reinforced toughening, as well as texture toughening. Based on the various toughening mechanisms in MAX phase, models of SiC particles and fibers toughening Ti_3_SiC_2_ are established to predict and explain the toughening mechanisms. The modeling work provides insights and guidance to fabricate MAX phase-related composites with optimized microstructures in order to achieve the desired mechanical properties required for harsh application environments.

## 1. Introduction

**Figure 1:**
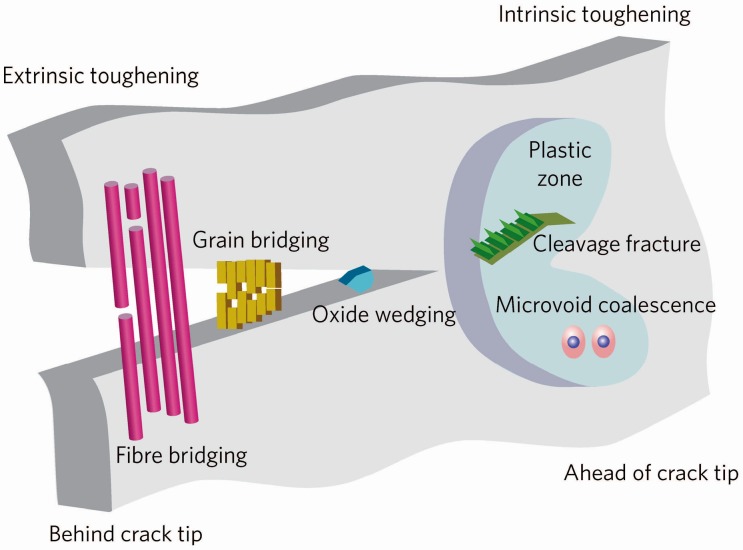
Schematic illustrating both intrinsic and extrinsic mechanisms of toughening mechanisms associated with crack extension [13].

Conventional ceramic materials mainly include glasses, structural clay products, whitewares, refractories, abrasives, cement and the newly developed advanced ceramics. Ceramic materials offer many distinct advantages such as excellent corrosion, wear resistance, high hardness and stiffness, chemical inertness, high-temperature characteristics as well as low density, which make them attractive for applications such as industries [1,2,3,4], medical [5], fuel constituents [6,7] and daily life [8], ranging in size scale from micro-electromechanical systems through large aerospace components. However, ceramic materials are severely limited in various fields of human activities by their mechanical properties [9,10,11]. The Achilles heel of ceramic materials is the low reliability, which is given by the disposition of inherent brittleness to catastrophic fracture with very low energy absorption. One of the macroscopic properties that characterizes this disposition of a ceramic is the fracture toughness (K_IC_) which is the resistance of ceramics to incipient cracking or defect generation and the microstructural mechanisms that are the source of such resistance [1]. The fracture toughness can be strongly influenced by microstructure or by the using of various reinforcements. There are two fundamentally different approaches: flaw control and toughening [12]. The toughening mechanisms have been well documented and can be conveniently considered to involve either intrinsic toughening mechanisms or extrinsic toughening mechanisms (Figure 1) [13]. The intrinsic toughening is an inherent property of the material, which is induced essentially from plasticity and enhances a material’s inherent damage resistance. Thus, it is active irrespective of crack size and geometry. The intrinsic toughening is the primary source of fracture resistance in ductile materials [13]. In contrast, most structural ceramics are essentially impossible to be tough intrinsically due to the absence of mobile dislocation activity [13,14] and the extrinsic toughening is the primary source of toughening in ceramics which act primarily behind the tip to impede crack advance. Extrinsic toughening acts to lower the local stress and strain fields at the crack tip without the inherent fracture resistance of the material changed; as it depends on the presence of a crack, it affects only the crack-growth toughness, specifically through the generation of a rising R-curve [13]. So far, a number of extrinsic toughening methods have been developed: transformation toughening, dispersed ductile/brittle particle toughening (microcracking toughening), fiber/lamella bridge toughening [15], whisker toughening [16], the frictional interlocking of grains during intergranular fracture in monolithic ceramics and complex structure toughening etc. Intergranular fracture is generally an essential requirement here for the operation of these mechanisms.

MAX phases are a group of new nanolayered ceramics with a general composition of M_*n*+1_AX_*n*_ [17,18,19,20,21,22] where M is an early transition metal, A is IIIA or IVA element, and X = C or N (*n* = 1~7). Until now, more than 50 M_2_AX phases (Ti_2_AlC, Cr_2_AlC, Ti_2_SnC etc.), 6 M_3_AX_2_ phases (Ti_3_SiC_2_, Ti_3_AlC_2_ etc.) and 8 M_4_AX_3_ phases (Ti_4_AlN_3_, Nb_4_AlC_3_, etc.) have been discovered and investigated along with many possible solid solution permutations and combinations [18]. In the unit cell of MAX phase, the M and X atoms form two common edges, M_6_X tetrahedron with the stronger covalent bond which is separated by A atomic planes, and the link between A atomic planes and M_6_X tetrahedron is weaker due to the M–A metallic bond. Due to such unique nanolaminated structure, the MAX phases are able to combine metal and ceramic materials demonstrating high thermal and electrical conductivities, excellent machinability and high-temperature oxidation resistance. Recently, MAX phases have attracted much attention due to their special qualities and potential applications in sensors, radiation hardened in nuclear energy and electrochemical energy storage systems [18,19,20,21]. In this content, enhancement of the reliability of these nanolayered MAX phases with desired mechanical properties becomes critical.

The present work gives a critical review of toughening mechanisms for the nanolayered MAX phases. Based on the present toughening work for MAX phases, microstructure-based models were established for a better understanding of the toughening mechanisms of these ceramics with nanolayered structures. Finally, an outlook of future directions for the toughening of these nanolayered ceramics is also provided.

## 2. Toughening in Nanolayered MAX Phases

The fascinating MAX phase ceramics have gained increasing attention due to their fruitful potential applications in industry. Great efforts have been made to investigate the fracture toughness and its enhancement of MAX phases [23]. The toughening mechanisms in MAX phases can be categorized into four basic types: particle toughening, phase-transformation toughening, fiber-reinforced toughening, and texture toughening.

### 2.1. Particle Toughening

The particle toughening is a general concept that refers to impending crack growth by dispersion particles such as TiC [24], Al_2_O_3_ [25], ZrC [26], BN [27,28] and carbon nanotubes (CNTs) [29] etc. Unfortunately, these brittle particle reinforcements can modestly enhance the toughness of MAX phases due to lack of plasticity of ceramic particles, and the most common activated toughening mechanisms are crack deflection and crack bridging.

**Figure 2:**
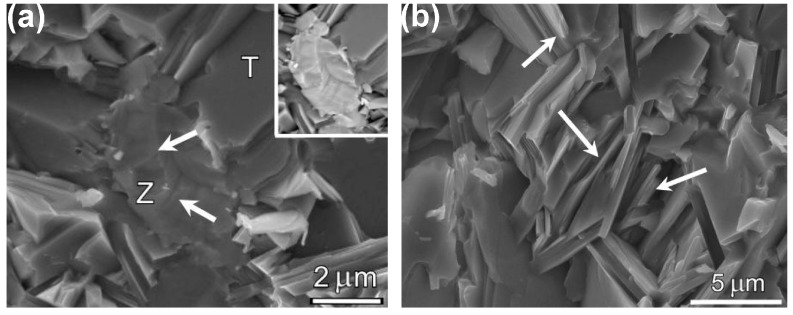
SEM micrographs of the fracture surface of the 20 vol % ZrC/Ti_3_AlC_2_ composite. (**a**) Interface between Ti_3_AlC_2_ and Zr, the inset: Backscattering image of area pointed with the white arrow; The Letters “T”, and “Z” represent Ti_3_AlC_2_, and ZrC, respectively; (**b**) A crack crossing through Ti_3_AlC_2_ grain and ZrC particle [26].

ZrC particles-reinforced Ti_3_AlC_2_ composites were obtained by in situ reactive hot-pressing at 1500 °C under a pressure of 30 MPa for 2 h in Ar using Ti, Al, graphite and ZrC powders as starting materials [26], and the fracture toughness of 20 vol % ZrC/Ti_3_AlC_2_ composite could reach up to 11.5 ± 1.0 MPa·m^1/2^ while the fracture toughness of monolithic Ti_3_AlC_2_ was 7.8 ± 0.4 MPa·m^1/2^. As shown in Figure 2, the fracture toughness improvement could be ascribed to energy consuming by ZrC particles and residual stresses in the ZrC/Ti_3_AlC_2_ composite derived from the cooling process in the composite preparation.

**Figure 3:**
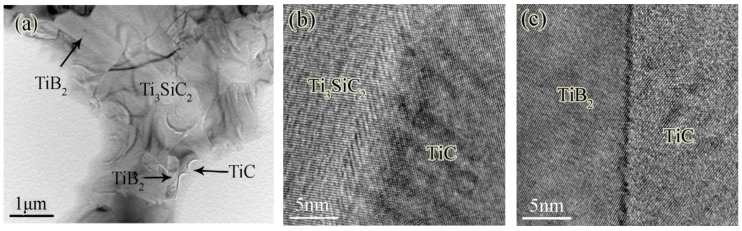
(**a**) TEM micrograph of the (TiB_2_+TiC)/Ti_3_SiC_2_ composite with 10 vol % TiB_2_; (**b**) HRTEM image of interface structure for Ti_3_SiC_2_ and TiC; (**c**) HRTEM image of interface structure for TiB_2_ and TiC [24].

A (TiB_2_ + TiC)/Ti_3_SiC_2_ composite with higher fracture toughness was obtained by hot-pressing at 1500 °C under a pressure of 25 MPa for 2 h in Ar atmosphere by applying TiH_2_, Si, graphite and B_4_C powders as starting materials [24]. The microstructures of the (TiB_2_ + TiC)/Ti_3_SiC_2_ composites (10 vol % TiB_2_) characterized by high-resolution transmission electron microscope (HRTEM) demonstrated clean and clear grain boundaries between the Ti_3_SiC_2_ matrix and reinforcements (Figure 3a,b). The fracture toughness of the as-prepared composites was higher than the pure Ti_3_SiC_2_ ceramic and other Ti_3_SiC_2_-matrix composites [30]. The fracture toughness of the (TiB_2_ + TiC)/Ti_3_SiC_2_ composites increased from about 9 MPa·m^1/2^ for the composites with 5 vol % TiB_2_ reinforcements to 9.55 MPa·m^1/2^ for the one with 10 vol % TiB_2_ reinforcements. However, further increasing TiB_2_ volume content up to 20 vol % resulted in a reduction of the fracture toughness of the composites [24]. The improvement of the fracture toughness of the composites was due to the very close interatomic distance of close-packed plane of atoms for TiB_2_ (0.3028 nm) and TiC (0.3055 nm), resulting in a coherent interface with strong binding energy (Figure 3c), which would lead to transgranular fracture and increase fracture energy as well as the residual compressive stress caused by the thermal expansion coefficient mismatch, which could increase the crack propagation resistance [24].

### 2.2. Whisker- and Fiber-Reinforced Toughening

In the whisker- and fiber-reinforced ceramic composites, the reinforcements could provide a ceramic matrix with large strains before failure and maintain themselves intact. However, limited work has been performed to investigate the toughening behaviors of fiber- or whisker-reinforced MAX phases due to the high reactivity between the fibers and MAX phase matrix. Most of the work focused on the fabrication of the whisker or fiber/MAX phase composites and the main fibers include SiC fiber [31,32,33], carbon fiber [34] and Al_2_O_3_ fiber [35,36].

**Figure 4:**
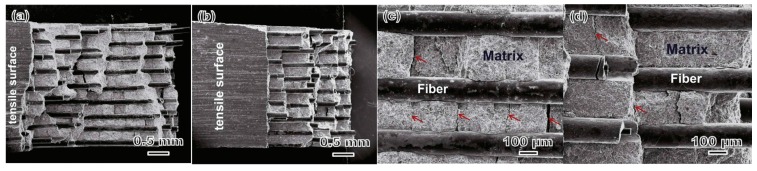
FE-SEM images of the fracture surfaces of (**a**,**c**) composites prepared at 1250 °C and (**b**,**d**) composites prepared at 1300 °C; the images show a step-like fracture model (a,b) and multiple transversal cracks in the matrix (c,d) [32].

The reactivity of Ti_2_AlC or Ti_3_SiC_2_ powders with uncoated SiC fibers at the temperature up to 1550 °C were evaluated [31]. The results indicated that the uncoated SiC fibers could be used as reinforcement in Ti_3_SiC_2_ but not in Ti_2_AlC, attributing to the reactivity that no apparent reaction occurred between Ti_3_SiC_2_ and SiC fibers, while Ti_2_AlC could react with SiC fibers to form Ti_3_(Al_1−*x*_Si_*x*_)C_2_, TiC and Al_1+*x*_Ti_1−*x*_ alloy. To reduce the reaction between the SiC fibers and Al-contained MAX phase, a Ti barrier layer was applied between the SiC fibers and Ti_3_AlC_2_ which could effectively hinder the inward diffusion of Al from the Ti_3_AlC_2_ matrix [32,33]. In the SiC_f_/Ti_3_AlC_2_ composites prepared at 1250 and 1300 °C, only minor phases of Ti_5_(Al,Si)_3_ alloy and TiC, as well as Ti_2_AlC were detected as interfaces. Compared to the monolithic Ti_3_AlC_2_ bulk, the load-displacement curves of as-prepared composites showed a noncatastrophic failure and a step-like fracture model was observed in the fracture surfaces using a field emission scanning electron microscope (FE-SEM, Figure 4) which indicated an improvement of the toughness [32].

Furthermore, Ti_2_AlC matrix loaded with two type of Al_2_O_3_ fibers (20 vol %) was obtained by spark plasma sintering (named as Ti_2_AlC/720f and Ti_2_AlC/610f) [36]. The fracture experiments and the post-mortem analysis of the fracture surface by SEM revealed that kinking along with intergranular cracking and delamination played an important role in deformation of Ti_2_AlC. The dynamic fracture toughness (5.46 MPa·m^1/2^) was higher than the quasi-static value (4.03 MPa·m^1/2^) by approximately 35%, and the fracture toughness of the composites reduced when increasing the temperature.

### 2.3. Transformation Toughening

Transformation toughening in MAX phases can be traced back to 2007 [37,38]. A 20 wt % ZrO_2_/Ti_3_AlC_2_ was obtained by uniaxial hot-pressing at 1450 °C under a pressure of 20 MPa for 2 h in vacuum and the measured fracture toughness of 20 wt % ZrO_2_/Ti_3_AlC_2_ composite was 6.8 MPa·m^1/2^, while the fracture toughness of monolithic Ti_3_AlC_2_ was 4.6 MPa·m^1/2^ [37]. The SEM observations of the fracture surface indicated that the fracture toughness improvement can be ascribed to the compressive stress in the matrix generated by the ZrO_2_ particles transformation process.

In the 3 mol % yttria stabilized tetragonal zirconia ceramics (3Y-TZP) reinforced Ti3SiC2 composites prepared by spark plasma sintering (SPS) at 1300 °C, the fracture toughness could reach 11.94 MPa·m^1/2^ with 30 vol % 3Y-TZP additions [38]. Some amount of m-ZrO_2_ phase could be found on the fracture surface indicating that the phase transformation of T-ZrO_2_→M-ZrO_2_ occurred during the fracture [38]. On the other hand, transformation toughening is not an exclusive toughening mechanism in the zirconia toughened composites (ZTCs). The second phase formed by phase transformation could toughen the matrix through multiple toughening mechanisms to a different degree, such as crack deflection and bridging [39].

### 2.4. Texture Toughening

Texture is also an effective approach for toughening of MAX phases ceramics [40,41,42,43,44,45,46]. So far, slip casting in a strong magnetic field coupled with spark plasma sintering was applied to produce textured MAX phases.

**Figure 5:**
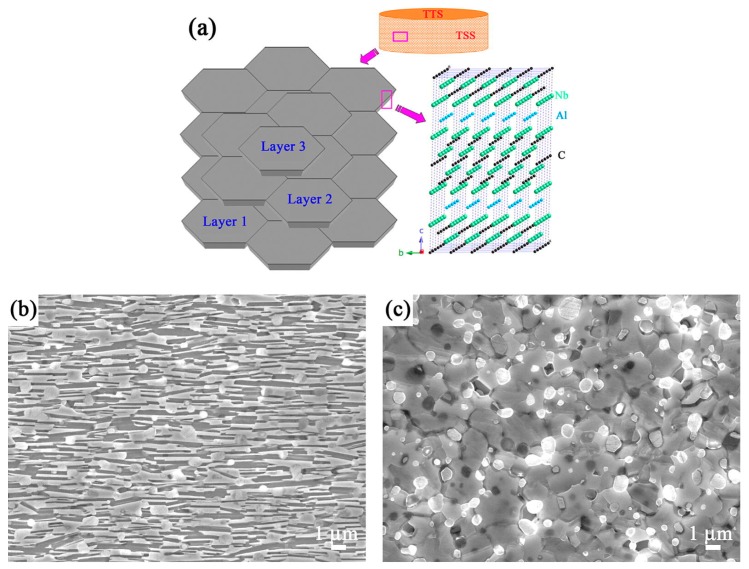

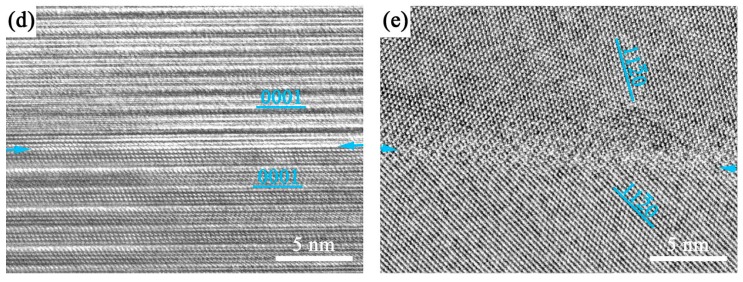
(**a**) Schematic diagram of tailored dense bulk nanolayered Nb_4_AlC_3_ ceramic, showing the orderly stacking of grains whose *c*-axes are perpendicular to the textured top surface; (**b**,**c**) SEM micrographs of etched textured side and top surface; (**d**,**e**) High-resolution transmission electron microscope atomic images of grain boundaries (indicated by arrows) observed from the textured side and top surface directions [40].

**Figure 6:**
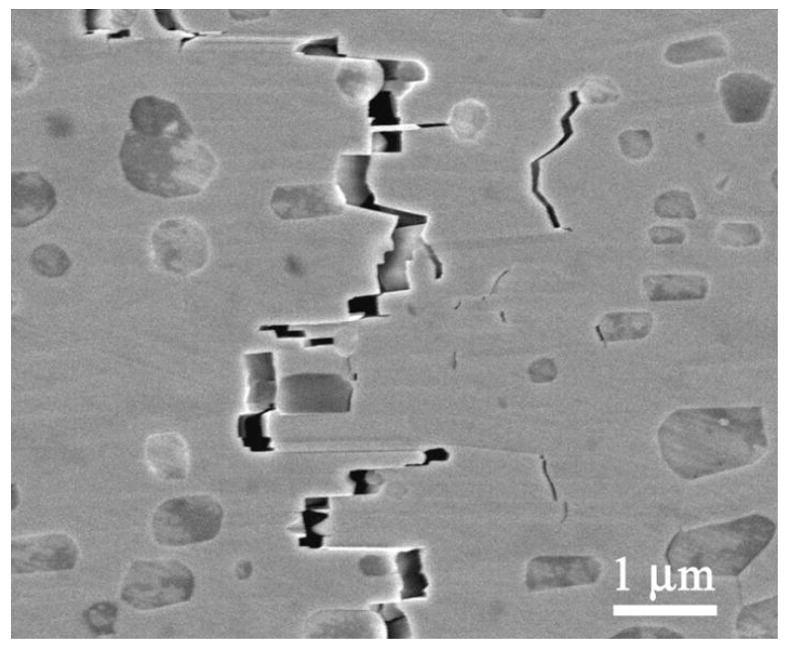
SEM micrographs of the in situ crack propagation of textured Nb_4_AlC_3_ [40].

Textured nanolayered Nb_4_AlC_3_ ceramic with a shell-like microstructure was fabricated by strong magnetic field alignment technology followed by spark plasma sintering [40]. After sintering, the tailored microstructures were schematized and analyzed by SEM and TEM observations (Figure 5). Figure 6 shows that the texture microstructure can activate the toughening mechanisms of crack deflection, through grain pull-out and bridging in Nb_4_AlC_3_ ceramic. Consequently, the fracture toughness of the textured Nb_4_AlC_3_ ceramic was extremely high and increased from 7.1 MPa·m^1/2^ for the as-grown ceramic to 17.9 ± 5.16 and 11.49 ± 1.38 MPa·m^1/2^ along the directions of parallel and perpendicular to the *c*-axis direction, respectively [40]. Similar work has been reported on other 211 and 312 MAX phases in a spark plasma facility and the fracture toughness of Ti_2_AlC increased from 6.0 MPa·m^1/2^ in the as-sintered ceramic to 7.9 MPa·m^1/2^ and 6.5 MPa·m^1/2^ parallel and perpendicular to the loading direction, respectively, in the deformed ceramic [42].

## 3. Toughening Models for MAX Phases

The toughening mechanisms in ceramics can be understood and predicted by the modeling work which can contribute insights and theoretical instructions for material design, process development and optimization. Many approaches were applied and developed for ceramic toughening mechanism simulations such as finite element method (FEM), first principles calculation (FPC) [47,48] and the empirical electron theory (EET) of solids and molecules [49,50,51,52].

**Figure 7:**
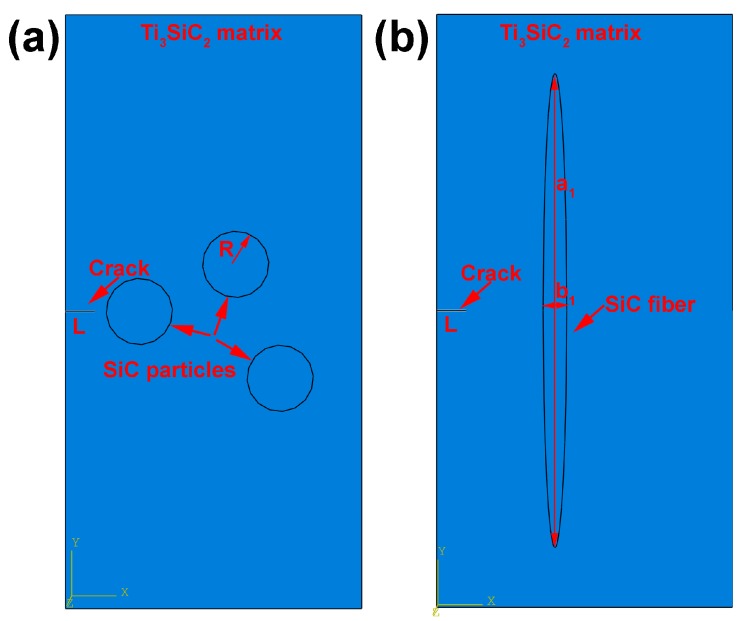
3D finite element model (3D-FEM) of SiC-reinforced Ti_3_SiC_2_ MAX phase with a crack on the left, (**a**) SiC particle (**b**) SiC fiber with a length–diameter ratio is 20:1.

For a comprehensive understanding of the toughening mechanisms in these nanolayered MAX phase ceramics, SiC particle and fiber-reinforced Ti_3_SiC_2_ composites were selected as models and simulated using a primary 3D finite element model (3D-FEM), which is schematically represented in Figure 7. The main material parameters used in 3D-FEM are listed in Table 1. For the SiC fiber toughening Ti_3_SiC_2_ MAX phase, the elliptical SiC fibers with a length–diameter ratio of 20:1 was applied in 3D-FEM.

**Figure 8:**
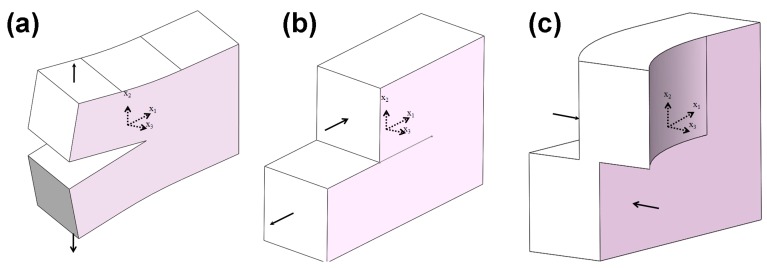
Schematic of the basic fracture modes under three-dimensional cracks: (**a**) Mode I (opening); (**b**) Mode II (shearing); (**c**) Mode III (tearing).

In fracture mechanics, there are three types of basic fracture modes, as shown in Figure 8: Mode I Opening model, Mode II Shearing mode and Mode III Tearing mode, where (*x*_1_, *x*_2_, *x*_3_) is the local Cartesian coordinate system centered at the crack front with the *x*_1_-axis perpendicular to the crack front, the *x*_2_-axis perpendicular to the crack plane, and the *x*_3_-axis along the crack front. In those modes, the stress-intensity factor, *K*, is a parameter describing the field of the crack tip which only correlates to the loads and geometries. In the present study, Mode I, the opening mode, was selected for SiC particle and fiber-reinforced Ti_3_SiC_2_ composites where the stress-intensity factor, *K*_I_, can be expressed by the following formula and be obtained from the extended finite element method (EFEM) by using an ABAQUS/CAE 2016 software package.

(1) $$K_{I}=\lim_{r\to 0}\sigma_{22}(x_{1}=a+r,x_{2}=0)\sqrt{2\pi r}$$

In the formula, σ_22_ is the biaxial stress in the vicinity of the crack tip, *x*_1_ and *x*_2_ are Cartesian coordinates at *x*_1_-axis and *x*_2_-axis, respectively, *a* is the crack length and *r* is the radius for a 3D penny-shaped crack.

### 3.1. SiC Particle-Reinforced Ti_3_SiC_2_ MAX Phase

**Figure 9:**
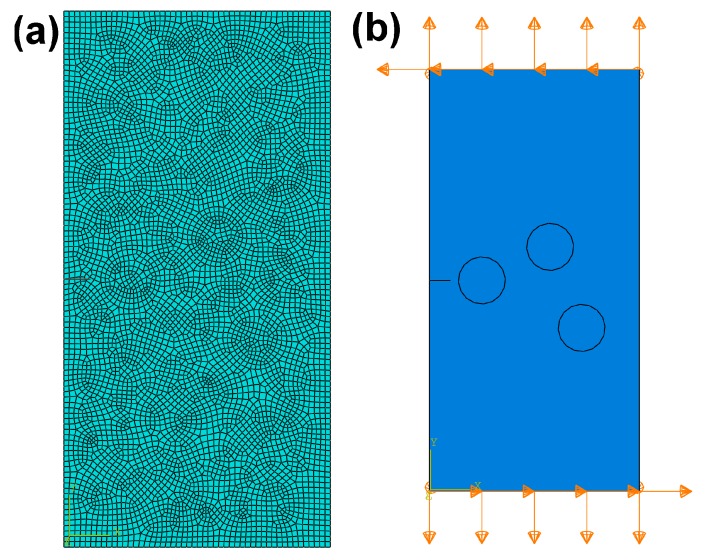
(**a**) Finite element mesh and (**b**) boundary conditions for the composite of SiC particles-reinforced Ti_3_SiC_2_.

**Figure 10:**
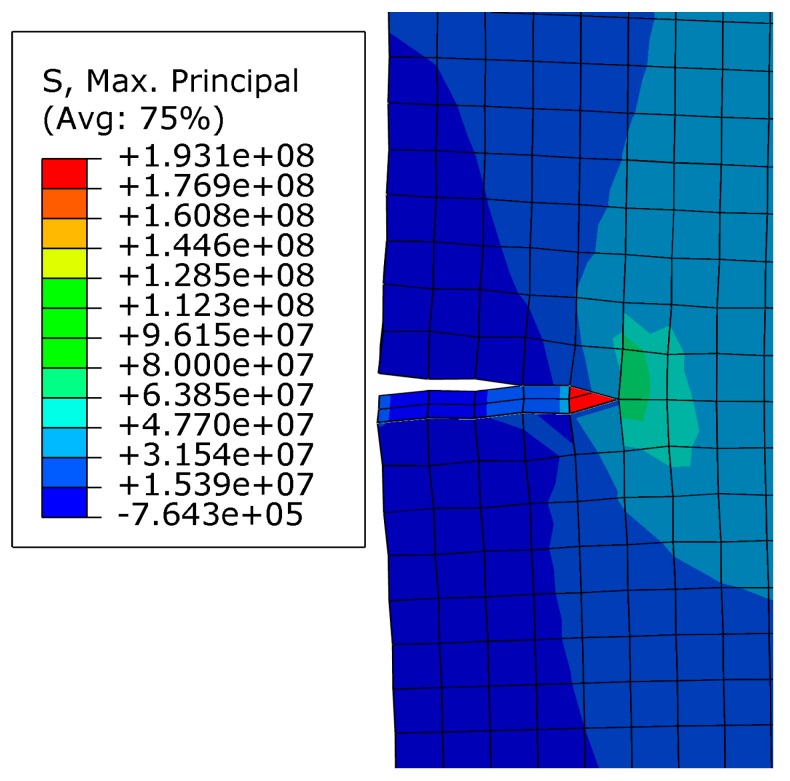
Predicted maximum principal stress distribution at the onset of crack propagation for the composite of SiC particle-reinforced Ti_3_SiC_2_.

Figure 9a represents the finite element mesh for the composite of SiC particle-reinforced Ti_3_SiC_2_ matrix, and Figure 9b shows the boundary conditions applied on the top and bottom sides of the geometry respect to the *X* axis and *Y* axis respectively. The geometry displacement at the *X* axis direction is constrained to zero. Figure 10 illustrates the maximum principal stress distribution at the onset of crack propagation predicted by the fracture model for the composite with 6.8 vol % SiC reinforcement. The corresponding value of the stress-intensity factor, *K*_I_, obtained from this simulation is 6.26 MPa·m^1/2^. As shown in Figure 10, the SiC particles can prevent crack from propagating and lead to stress concentration at the crack tip and the stress-intensity factor *K*_I_ can be considered as a critical factor for fracture toughness (*K*_I_ = *K*_IC_ = 6.26 MPa·m^1/2^), which demonstrates a sufficient reinforcement to enhance the toughness of monolithic Ti_3_SiC_2_ MAX phase [30].

### 3.2. SiC Fiber-Reinforced Ti_3_SiC_2_ MAX Phase

**Figure 11:**
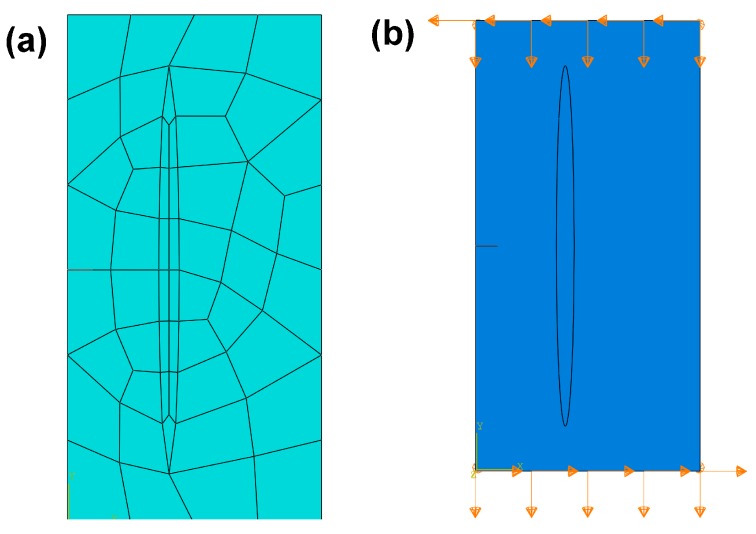
(**a**) Finite element mesh; (**b**) boundary constraint for the composite of SiC fiber-reinforced Ti_3_SiC_2_.

**Figure 12:**
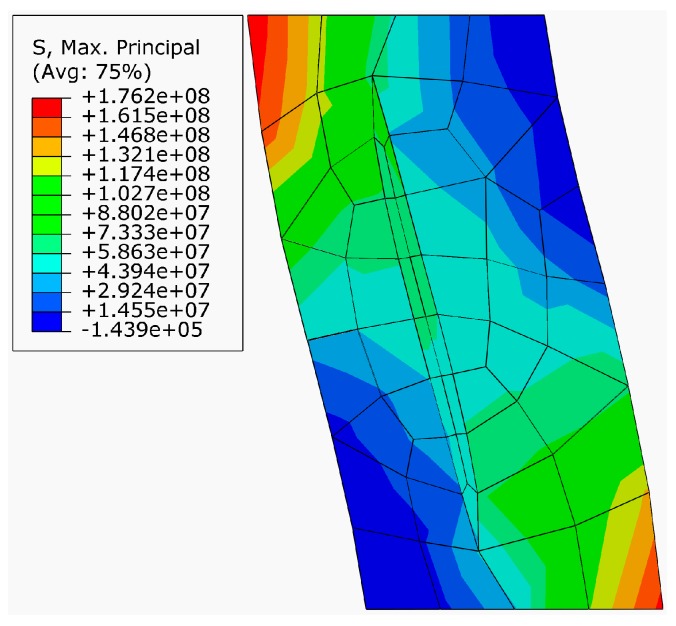
Predicted maximum principal stress distribution at the onset of crack propagation for the SiC fiber-reinforced Ti_3_SiC_2_ composites.

Similar to SiC particle-reinforced Ti_3_SiC_2_ MAX phase, Figure 11 shows the finite element mesh and boundary constraint for the SiC fiber-reinforced Ti_3_SiC_2_ composite. In the finite element mesh (Figure 11a), the seed point distribution on the inner ellipse SiC fiber edge are 16 points and the seed distribution on the cubic edge of Ti_3_SiC_2_ matrix are 6 points at its high dimension, 4 points at its long dimension and 1 point at its wide dimension. For the boundary constraint, as shown in Figure 11b, the displacement of the top of Ti_3_SiC_2_ matrix constraint to −4 × 10^5^ mm in the U_1_ direction, −2 × 10^5^ mm in the U_2_ direction and 0 mm in the U_3_ direction, the displacement of the bottom of Ti_3_SiC_2_ matrix constraint to 4 × 10^5^ mm in the U_1_ direction, −2 × 10^5^ mm in the U_1_ direction and 0 mm in the U_3_ direction. Consequently, Figure 12 shows the predicted maximum principal stress distribution at the onset of crack propagation for the proposed SiC fiber-reinforced Ti_3_SiC_2_ composites with 5 vol % reinforcements_._ The predicted maximum principal stress is 176.2 MPa. The higher the maximum principal stress, the stronger the fiber can prevent crack from initiating. The stress-intensity factor *K*_I_ computed from 3D-FEM method is 12.65 MP·m^1/2^ indicating a significant improvement in the toughness of the Ti_3_SiC_2_ MAX phases.

The successful prediction of toughness of the SiC particle and fiber-reinforced Ti_3_SiC_2_ using 3D-FEM can provide insight and valuable guidance to the experimental work to fabricate MAX phase-based composites with optimized microstructures to obtain the desired mechanical properties. However, more modeling prediction should be done to further optimize the microstructures of the composites to achieve the required properties for their applications.

## 4. Conclusions

This paper reviews the toughening mechanisms for a family of nanolayered MAX phases and the related composites. Based on the experimental results, SiC particle and fiber toughening MAX phase models were established for a better understanding of the toughening mechanisms and prediction of the improvement of the toughness, which may contribute with insights and theoretical instructions for material design, process development, and optimization. Meanwhile, it also opens many perspectives in toughening those nanolayered ceramics:

(1) To apply these nanolayered MAX phases as higher performance and reliable structural components, a further tailoring of the microstructure should be done to enhance both strength and toughness. Through additional microstructure modification e.g., by grain size control, it is probable that the flexural strength or fracture toughness can be further enhanced.
(2) For the fiber toughening MAX phases, more work should be done to optimize the interface between MAX phase and fibers, e.g., by selecting different fibers which could be phase equilibrium with MAX phase during the high-temperature processing or by new processing methods that can consolidate the composites with fast densification technology to reduce or avoid the reaction between the fibers and MAX phase matrix.
(3) The modeling work presented here is just a first attempt to predict the improved toughness of the MAX phase-based composites by using 3D-FEM. However, more modeling parameters such as selection of proper reinforcements, the volume fraction of reinforcement as well as size and dimensions of the reinforcements etc. need to be further investigated and refined, which may provide valuable theoretical guidelines for material design, process development, and optimization.

Success in addressing these points may expand MAX phases and related composites to numerous structural and high-temperature applications.

## Figures and Tables

**Table 1:** Main material parameters in 3D-FEM.

Composites	Materials	Young’s Modulus (GPa)	Poisson’s Ratio	Dimension	Volume Fraction (%)	Half Crack Length (mm)
SiC-Ti_3_SiC_2_ system	Ti_3_SiC_2_ matrix	333	0.2	0.05 × 0.1 × 0.002 mm^3^	93.2	0.005
	SiC particle	440	0.14	*R* = 0.006 mm	6.8	
SiC-Ti_3_SiC_2_ system	Ti_3_SiC_2_ matrix	333	0.2	0.05 × 0.1 × 0.002 mm^3^	95	0.005
	SiC-Fiber	450	0.14	*a*_1_ = 0.08 mm *b*_1_ = 0.004 mm	5.0	
